# Nucleic acids in medicinal plants and their pharmaceutical potential

**DOI:** 10.20517/evcna.2026.57

**Published:** 2026-09-18

**Authors:** Xiaoyan Qin, Fangwen Jiao, Hongzhi Qiao

**Affiliations:** ^1^Jiangsu Key Laboratory of Medicinal Substance and Utilization of Fresh Chinese Medicine, School of Pharmacy, Nanjing University of Chinese Medicine, Nanjing 210023, Jiangsu, China.; ^2^School of Medicine, Nanjing University of Chinese Medicine, Nanjing 210023, Jiangsu, China.; ^3^Jiangsu Engineering Research Center for Efficient Delivery System of traditional Chinese medicine (TCM), School of Pharmacy, Nanjing University of Chinese Medicine, Nanjing 210023, Jiangsu, China.

**Keywords:** Medicinal plants, nucleic acids, small RNAs, drug development

## Abstract

Medicinal plants, as a core constituent of traditional medicine, provide an essential foundation for preventing and treating human diseases. Research on their pharmacologically active constituents has long focused on secondary metabolites, including alkaloids, terpenoids, flavonoids, and other compounds. However, with the rapid advancement of molecular biology techniques, nucleic acids have gradually become a research focus as a novel class of active components in medicinal plants that possess both nutritional and regulatory functions. This paper systematically reviews the types, characteristics, extraction techniques, pharmacological actions, and mechanisms of nucleic acids (including coding and non-coding RNAs) in medicinal plants. It also discusses current challenges and future directions for medicinal plant-derived nucleic acids in new drug development, providing a theoretical reference and technical basis for in-depth research and industrial application in this field.

## INTRODUCTION

Medicinal plants, as a primary source of natural metabolites with specific pharmacological activities for humans, have long been a research focus^[1]^. In ancient civilizations such as China, India, and Egypt, the use of medicinal plants to treat various diseases dates back 5,000 years. Even in the current era of widespread modern pharmaceuticals, they remain widely used^[2]^, playing an essential role in disease prevention, treatment, and health maintenance. Traditionally, research on the active components of medicinal plants has primarily focused on secondary metabolites, such as alkaloids^[3]^, flavonoids^[4]^, quinones^[5]^, and terpenoids^[6]^, while nucleic acids have long been regarded solely as genetic material. These nucleic acids (including DNA, mRNA, siRNA, miRNA, and so on) carry plant cells’ genetic information and participate in key physiological processes such as gene expression, protein synthesis, and cell proliferation and differentiation. During processing or digestion, these nucleic acids are degraded into smaller molecules like nucleotides and deoxyribonucleotides, which are then absorbed and utilized by the human body (as shown in Figure 1). However, in recent years, researchers increasingly recognize that nucleic acids in medicinal plants may represent one of the key active components responsible for their pharmacological effects^[7]^.

**Figure 1:**
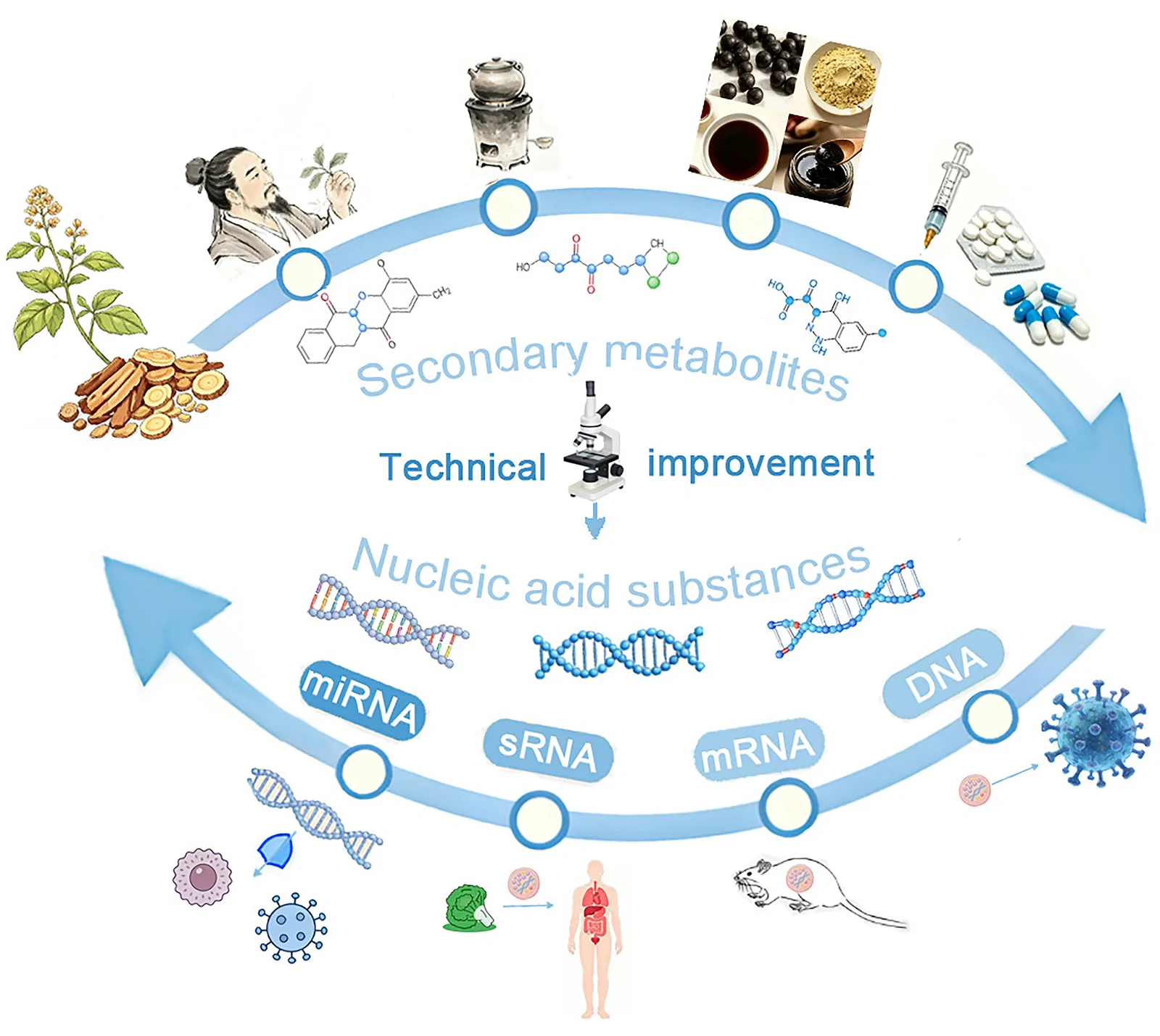
Research status of active substances in medicinal plants. sRNA: Small RNA; miRNA: microRNA; mRNA: messenger RNA.

Research on medicinal plant-derived nucleic acids as active components has gradually deepened, from basic discovery to functional verification. In 1998, Fire *et al*. discovered the phenomenon of double-stranded RNA interference. They found that exogenous double-stranded RNA introduced into *Caenorhabditis elegans* triggers gene silencing by binding to endogenous messenger RNA (mRNA) transcripts, and this inhibitory effect is markedly stronger than that induced by purified single-stranded RNA. This landmark finding revealed that nucleic acids serve not only as genetic carriers but can also participate in regulatory biological processes^[8]^. In 2011, Zhang *et al*. discovered that plant-derived small nucleic acids can enter human blood and tissues through dietary intake. Once internalized, these small nucleic acids influence physiological functions by regulating target gene expression, thereby exerting biological effects and, for the first time, confirming the cross-kingdom regulatory effect of plant-derived small RNAs in mammals^[9]^. In 2019, Huang *et al*. demonstrated that small nucleic acids can be fully extracted from various medicinal plants even after boiling decoction, which provided experimental support for the clinical application of medicinal plant-derived nucleic acids through oral administration^[10]^. Extensive research has now confirmed that nucleic acids show significant therapeutic efficacy and pharmaceutical potential in immune modulation^[11]^, antitumor activity^[12]^, antiviral effects^[13]^, and other areas. This review summarizes the types of nucleic acids in medicinal plants, their extraction techniques, pharmacological properties, mechanisms of action, and future research and development prospects, and it is intended to provide a scientific reference for further in-depth investigations in this emerging field.

## NUCLEIC ACIDS IN MEDICINAL PLANTS

Medicinal plants contain a rich repertoire of nucleic acids, including both DNA and RNA. As functional molecules with remarkable regulatory potential and pharmaceutical value, RNAs have emerged as the core focus of current research on medicinal plant-derived bioactive nucleic acids. Based on their protein-coding capacity, cellular RNAs can be broadly classified into coding RNAs (mRNA) and non-coding RNAs (ncRNAs)^[14]^. NcRNAs refer to RNA molecules that do not encode proteins, and according to their length, they can be divided into small ncRNAs (sncRNAs, < 200 nt) and long ncRNAs (lncRNAs, > 200 nt). Among these, small regulatory RNAs, typically composed of 20-40 nucleotides, have attracted extensive attention and become key targets for identifying novel active components from medicinal plants^[15]^.

Current research primarily focuses on two major sRNAs: small interfering RNA (siRNA) and microRNA (miRNA)^[16]^. RNA interference (RNAi) represents an evolutionarily conserved post-transcriptional gene regulatory mechanism. Since its discovery in *Caenorhabditis elegans* in 1998, it has reshaped the landscape of drug development^[8,17]^. siRNAs selectively degrade targeted mRNAs before translation, preventing their translation into amino acids and subsequent protein synthesis. By silencing the gene encoding these mRNAs, it blocks the production of abnormal or harmful proteins^[18]^. In contrast, miRNAs modulate gene expression by base-pairing with target mRNAs, leading to suppressed translation or accelerated degradation of target transcripts at the post-transcriptional level. This regulatory mode ultimately shapes various physiological processes in organisms^[14]^.

However, it is noteworthy that in contrast to the extensive mismatches observed for most animal miRNA binding sites, many plant miRNAs bind their targets with perfect or near-perfect complementarity^[19]^. Accumulating evidence suggests that plant-derived miRNAs from medicinal plants can survive in the human body and exert pharmacological effects via cross-kingdom regulation of human gene expression^[20]^. Furthermore, combined comparative genomic analysis revealed that more than 60% of human protein-coding genes harbor miRNA binding sites maintained by evolutionary purifying selection^[21]^, providing a theoretical basis for multi-target regulation by exogenous plant-derived miRNAs. Accordingly, plant-derived miRNAs may participate in diverse human physiological and pathological processes and represent promising novel active constituents from medicinal plants^[22,23]^. These discoveries have sparked widespread research interest in medicinal plant-derived miRNAs. Meanwhile, imbalances in endogenous miRNA expression are a key factor in the onset and development of numerous diseases, including malignant tumors. Exogenous miRNA supplementation to restore regulatory effects offers novel therapeutic strategies for disease treatment. Furthermore, based on the property that a single miRNA can bind multiple target sequences, a single miRNA can simultaneously act on multiple critical nodes in disease pathways. This feature suggests the therapeutic potential of miRNA therapy against various diseases^[14]^. Accordingly, this review focuses on recent research progress on miRNAs derived from medicinal plants, and also supplements the research status of other ncRNAs and functional DNA fragments in medicinal plants.

### Distribution of miRNAs in medicinal plants

As the most representative bioactive nucleic acid component in medicinal plants, miRNA has characteristic distribution patterns that underlie studies of its biological function. Since the first report of miRNAs in 1993^[24]^, by 2026, the miRBase 22.1 database has cataloged a total of 48,885 mature miRNA sequences from 271 species, including numerous medicinal plant and animal species. Medicinal plant materials contain abundant miRNAs that play crucial roles in regulating plant physiology. These miRNAs show clear tissue- and developmental-stage specificity in medicinal plants, and their expression levels are closely related to secondary metabolite synthesis and the pharmacological activity of medicinal plants. For instance, high-throughput sequencing of miRNAs extracted from the roots, stems, and leaves of *Rehmannia glutinosa* revealed 89 conserved miRNAs and six novel miRNAs. KEGG (Kyoto Encyclopedia of Genes and Genomes) enrichment analysis indicated that these miRNAs primarily function in transcriptional regulation, plant growth and development, and signal transduction. The expression level of miR156 in *Rehmannia glutinosa* roots is markedly higher than that in stems and leaves. By targeting squamosa promoter-binding protein-like (SPL) genes, high miR156 levels can prolong the development of tuberous roots and thereby indirectly influence the biosynthesis and accumulation of catalpol, the primary bioactive constituent of *Rehmannia glutinosa*, which is one of the important reasons for the high pharmacological activity of *Rehmannia glutinosa* roots^[25]^. The identification status of common medicinal plant-derived miRNAs is shown in Table 1.

**Table 1:** Identification status of miRNAs in common medicinal plants

**Medicinal plants**	**Number of identified miRNAs**	**Ref.**
*Pinellia ternata*	54 miRNAs	[26]
*Pinellia pedatisecta*	101 miRNAs	[27]
*Xanthium strumarium *L.	1,222 miRNAs	[28]
*Panax notoginseng*	368 miRNAs	[29]
*Aquilaria sinensis*	74 miRNAs	[30]
*Panax ginseng C.A.Meyer*	280 miRNAs	[31]
*Rehmannia glutinosa*	201 miRNAs	[32]
*Lilium lancifolium* Thunb	82 miRNAs	[33]
*Elettaria cardamomum Maton*	161 miRNAs	[34]
*Papaver somniferum*	327 miRNAs	[35]
*Taxus mairei*	871 miRNAs 869 miRNA precursors	[36]
*Ganoderma lucidum*	168 miRNAs	[37]
*Panax ginseng*	71 miRNAs	[38]
*Salvia miltiorrhiza *Bunge	492 miRNAs	[39]
*Lonicera japonica *	256 miRNAs	[40]

miRNAs: MicroRNAs.

### Extraction of miRNAs from medicinal plants

Extracting high-purity, high-integrity RNA from medicinal plants is a fundamental step for high-throughput sequencing, bioinformatics analysis, and pharmacodynamic evaluation. Currently, the primary method for extracting total RNA from plants relies on reagent kits. According to different reagents and principles, these methods are mainly divided into Trizol, RNAiso Plus, sodium dodecyl sulfate (SDS), and Cetyltrimethylammonium Bromide (CTAB)^[41]^ methods. However, the composition, structure, content, and distribution of RNA vary significantly between different plant species, among different tissues in the same plant, and even across developmental stages of the same tissue or organ^[42-44]^. Consequently, different extraction methods may yield remarkably different RNA quality from the same tissue sample. After processing and boiling, RNA content in medicinal plant slices may decrease. Certain plant tissues, rich in phenolic compounds, polysaccharides, or other unidentified secondary metabolites, can interfere with the extraction process, thereby affecting both RNA quantity and quality^[45]^. For instance, when extracting small RNAs from the tropical fruit papaya, using a lithium chloride (LiCl) extraction buffer yields small RNAs of poor quality and low quantity; it is more appropriate to use Trizol and CTAB together for extraction^[46]^. Therefore, when extracting RNA from medicinal plants, different methods are typically selected based on the species, origin, tissue type, and processing conditions of the dried medicinal materials, and targeted optimization schemes are formulated according to the characteristics of different medicinal plants. The principles, advantages, and disadvantages of commonly used methods are summarized in Table 2. The core challenges of miRNA extraction from medicinal plants are the interference of secondary metabolites (polyphenols/polysaccharides) and the degradation of RNA after thermal processing; thus, the optimal extraction method should be selected based on the plant tissue type and processing status, and combined with purification steps (e.g., LiCl precipitation) to improve RNA purity and integrity.

**Table 2:** Principles, advantages and disadvantages of different methods for plant total RNA extraction

**Extraction method**	**Principle**	**Advantages**	**Disadvantages**	**Ref.**
Trizol method	Utilizing the strong denaturing and oxidizing capacity of guanidine isothiocyanate or phenol in the extraction solution to break down cell membranes and denature proteins, thereby inhibiting the activity of endogenous RNase. This process simultaneously releases nucleic acids into the aqueous phase, enabling RNA recovery via precipitation	Simple operation and high RNA extraction integrity make it suitable for plant tissues with low secondary metabolite content	Polyphenolic compounds undergo oxidation during extraction, forming irreversible bonds with nucleic acid macromolecules. While chloroform extraction removes these polyphenolic compounds, it simultaneously causes RNA loss	[47]
SDS-phenol method	Using SDS and phenol can denature proteins, inhibit endogenous RNase activity, and break down cell membranes. This allows proteins, polysaccharides, and DNA in the solution to be separated into the organic phase, while RNA remains in the supernatant	Simple operation, short extraction time, capable of removing polysaccharides and phenolic compounds from samples	This process generates a high content of polyphenolic impurities, and the extracted plant total RNA cannot be used for reverse transcription experiments	[48]
SDS-LiCl method	The SDS-LiCl method uses high-concentration urea to denature proteins and inhibit ribonuclease activity, then selectively precipitates RNA with LiCl	For plant tissues rich in starch and other secondary metabolites, yielding high-yield, high-quality RNA free from protein contaminants, organic solvents, and other byproducts	RNA degradation may occur	[49]
CTAB method	CTAB is a cationic surfactant. In solutions with high ionic strength, CTAB forms complex precipitates with proteins, polyphenols, and neutral polysaccharides, allowing nucleic acids to dissolve in the aqueous phase, thereby achieving nucleic acid separation	High RNA purity, excellent integrity, and high success rate	65 °C causes severe structural damage to RNA, leading to its degradation and reduced yield	[50]
HB	Boric acid can form complexes with phenolic compounds via hydrogen bonding. DTT acts as a reducing agent, NP-40 prevents oxidation of phenolic substances, and PVP forms complexes with polyphenolic compounds. Residual phenolic substances are precipitated using LiCl, thereby separating them from RNA	High concentrations of borate inhibit oxidase enzymes, protecting RNA from interference by polyphenols. Additionally, proteinase K degrades phenolic compounds and secondary metabolite oxidases, resulting in RNA of the highest purity and generally good integrity	The required drugs are numerous, expensive, and time-consuming to extract, with complex procedures that make them susceptible to degradation by exogenous RNases	[51]
GT	Guanidine isothiocyanate acts as a potent denaturant, inducing structural changes in nucleic acid-protein complexes and effectively dissociating nucleoprotein-nucleic acid complexes. In combination with β-mercaptoethanol, it strongly inhibits RNase activity, breaks down cells, and rapidly releases nucleic acids. DNA is then removed via CsCl density gradient centrifugation, effectively yielding total RNA	Guanidine isothiocyanate strongly inhibits RNase activity in samples. The method is simple to perform, ensures high integrity, yields high output, requires minimal time, and is cost-effective	The extracted RNA has low purity and requires further purification	[52]
LiCl extraction method	This method denatures proteins with high-concentration urea, inhibits ribonuclease activity, and selectively precipitates RNA with LiCl	Suitable for extracting small amounts of tissue RNA from a large number of samples, with the advantage of being fast and straightforward	Minor DNA contamination and low RNA yield, with loss of small RNA fragments	[53]
Spin column-based RNA extraction	Guanidine hydrochloride breaks down samples and inhibits RNase activity; β-mercaptoethanol denatures proteins. RNA is specifically adsorbed onto the membrane in the spin column	Simple and rapid operation, safe, high product purity, ready for immediate use in downstream experiments	Most require manual operation, may cause membrane clogging, and have specific size requirements for RNA	[54]

PVP: Polyvinylpyrrolidone; RNase: ribonuclease; RNA: ribonucleic acid; DNA: deoxyribonucleic acid; CTAB: cetyltrimethylammonium bromide; SDS: sodium dodecyl sulfate; LiCl: lithium chloride; DTT: dithiothreitol; NP-40: nonidet P-40; GT: guanidine thiocyanate method; HB: hot boric acid method.

### Stability of miRNAs in medicinal plants

#### Stability of medicinal plant-derived miRNAs during steaming and cooking

Many medicinal plants need to be decocted before application; thus, partial plant miRNAs with pharmacological activity should maintain stability after high-temperature processing. Current studies support this notion and have obtained substantial quantitative data on the stability of medicinal plant-derived miRNAs during thermal processing. For instance, researchers identified 43 miRNAs from 71 miRNA families in this decoction using high-throughput sequencing and the research results showed that the average retention rate of miRNAs in ginseng decoction after boiling for 30 min was 65.2%, the retention rate after boiling for 60 min was 42.8%, and the retention rate after boiling for 90 min was 28.5%^[55]^, indicating that the retention rate of miRNAs decreases with the extension of boiling time and some miRNAs from medicinal plants can remain stable during soaking and boiling processes. In an analysis of miRNA concentrations in cooked corn feed, although levels decreased to 1/30 of those in fresh corn after boiling for 60 min, 18 corn miRNAs remained detectable across different corn feeds and even after severe puffing processing, indicating a certain degree of resistance to harsh cooking conditions^[56]^. Subsequent experiments with Viscum album (mistletoe) also confirmed the stability of plant-derived miRNAs (including miR-166a-3p, miR-159a, miR-831-5p, val-miR-218, and val-miR-11) during medicinal plant extraction, supporting that bioactive medicinal plant-derived miRNAs can persist in mammalian organisms^[57]^. Some representative examples are shown in Table 3.

**Table 3:** Stability of miRNAs under different treatment conditions

**Plant source**	**Processing conditions**	**Description**	**Ref.**
Maize	Harsh puffing treatment	The total miRNA content dropped to one-thirtieth of that in fresh samples, yet all 18 miRNAs remained detectable	[56]
*Viscum album* L. (European mistletoe)	Incubation at 100 °C for 30 min/intensive mechanical treatment/be exposed to RNase	Some miRNAs, such as miR-166a-3p and miR-159a, showed a significant decrease	[57]
Peanut, tomato, sorghum, cabbage, soybean, rice	Simulated digestive system: add gastric fluid and intestinal fluid (pH = 7.8) at a 1:1 ratio and incubate at 37 °C	Six plant miRNAs (miR-157a, miR-172a, miR-894, miR-159, miR-160, miR-168a) underwent severe degradation within 120 min	[58]
Fruits, vegetables, nuts, legumes, and cereals	Pressure-cooked for 5-30 min	miR-156e, miR-159, and miR-162 can still be detected after high-pressure heat treatment	[59]
*Lonicera japonica* (honeysuckle)	Boiled in water for 30 min	MIR-2911 was found to be largely intact	[60]
Artichoke	Boiled in sterile water for 15 min at 100 °C	Cooking reduced the total RNA content by approximately 39%	[61]
Artichoke	Salivary-gastric step (SG)/salivary-gastric-intestinal step (SGI)	No significant difference in RNA concentration between the 2 digestion steps, and the amount of RNA found after the digestive process was 20 times less than the amount of RNA in the raw samples	[61]
*Citrus sinensis*	Salivary nuclease incubation	There was almost no degradation of the miRNA after mixing with the food matrix	[62]

miRNA: MicroRNA; miRNAs: microRNAs; RNase: ribonuclease.

#### Stability of medicinal plant-derived miRNAs in the digestive tract and circulatory system

The miRNAs of medicinal plants mostly enter the human body through oral administration to exert their effects. Therefore, maintaining the stability of miRNAs in the body is an important prerequisite for them to function, this section further explores the stability maintenance mechanisms of medicinal plant-derived miRNAs in the human digestive tract and circulatory system.

From a biochemical perspective, exogenous biomolecules such as proteins, lipids, and nucleic acids are broken down into their most fundamental components - including amino acids, glycerol, nucleotides, and other minimal units - after entering the digestive tract for absorption. Accordingly, it has traditionally been believed that proteins, lipids, and nucleic acids lose their biological activity through digestion and degradation in the gastrointestinal tract upon entering the human body. However, recent studies have revealed that miRNAs can maintain stability in the digestive tract and circulatory system [Table 3], subsequently entering animals or humans to exert pharmacological effects^[63]^. These indicate that nucleic acid degradation pathways are not the only mechanism underlying their absorption.

As illustrated in Figure 2, from a chemical structural perspective, 2’-O-methylation at the 3’ end of plant miRNAs protects them, thereby enhancing their stability and enabling stable persistence in animal organisms^[64]^. Furthermore, the intrinsic base composition and spatial secondary structure of miRNAs are critical factors that may regulate their stability* in vivo*. Regarding base composition, the guanine-cytosine (G-C) content significantly influences a miRNA’s resistance to degradation; CG base pairs - stabilized by three hydrogen bonds - offer greater binding stability than the two hydrogen bonds found in adenine-uracil (A-U) pair. Consequently, medicinal plant-derived miRNAs with high CG content exhibit more robust base pairing, enabling them to effectively withstand cleavage by gastrointestinal nucleases and physicochemical disruption in body fluids^[65]^. Regarding spatial structure, miRNAs can form stable secondary structures - such as stem-loops and hairpins - through complementary base pairing. These folded conformations can shield the core nucleic acid sequence, limiting the binding sites available for nucleases and evading recognition and degradation by various hydrolases in digestive fluids and the circulatory system^[56,66]^.

**Figure 2:**
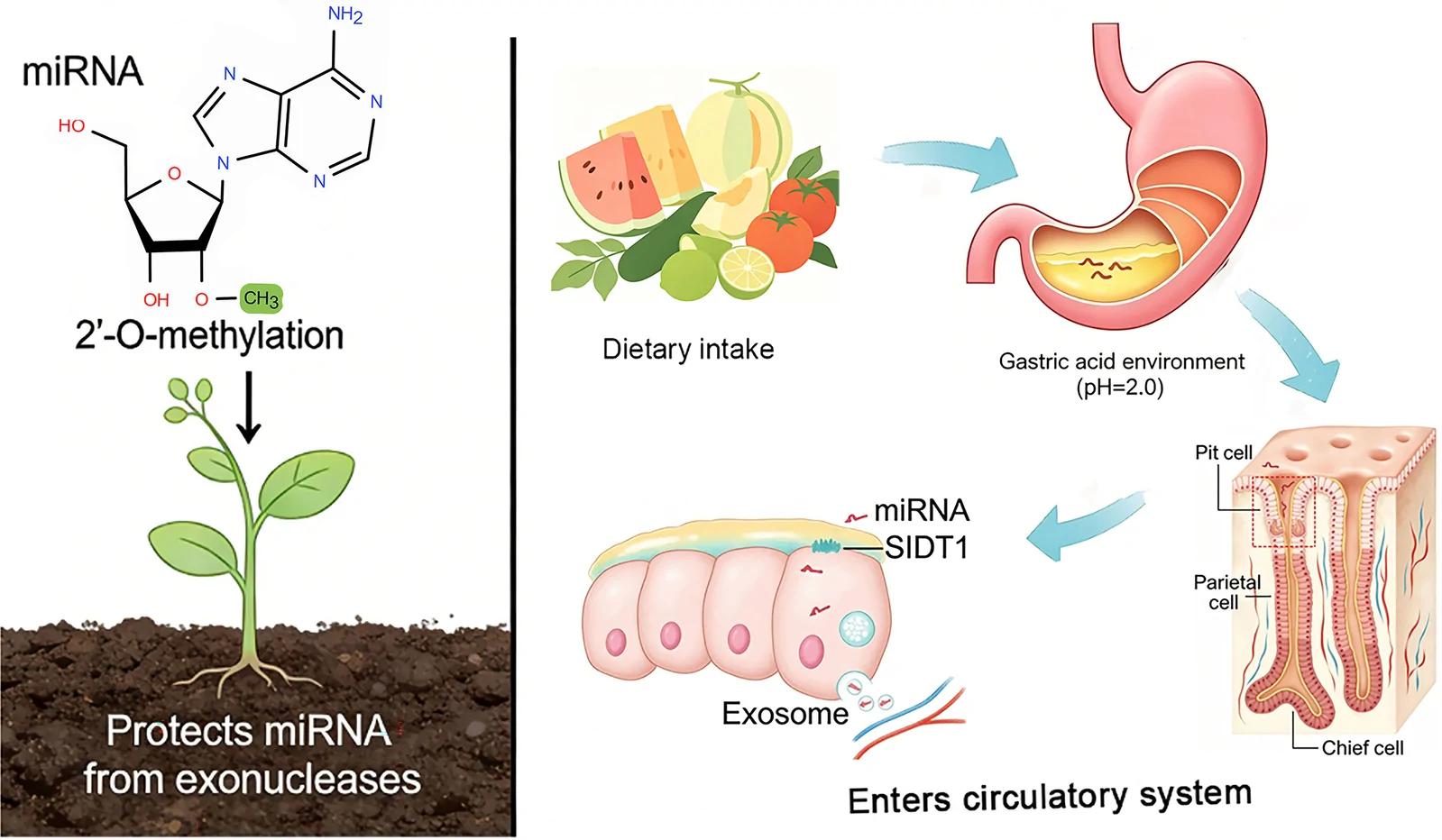
Stability maintenance mechanisms of medicinal plant-derived miRNAs in the digestive tract and circulatory system. SIDT1: SID-1 transmembrane family member 1; miRNA: microRNA.

Regarding transport pathways, researchers have uncovered unique absorption mechanisms for dietary miRNAs. In the stomach, dietary miRNAs are taken up by pit cells via the endogenous transporter SID-1 transmembrane family member 1 (SIDT1) and subsequently released within extracellular vesicles. These vesicles shelter miRNAs from degradation in circulation and facilitate subsequent cellular uptake, and this absorption process relies on low-pH conditions^[67,68]^. In addition, plant miRNAs exhibit markedly slower degradation rates than animal miRNAs under extreme gastric acid conditions (pH 2.0)^[64,69]^. Beyond this, vesicle encapsulation enhances miRNA resistance against harsh gastrointestinal stress^[70]^. Notably, plant-derived exosome-like nanovesicles offer superior protection of cargo miRNAs against ribonuclease (RNase) digestion, gastric acid, and intestinal alkaline environments compared with mammalian milk exosomes^[71]^. Consistent with in vitro degradation experiments, acerola-derived exosome-like nanovesicles (AELNs) confer significantly stronger shielding effects on miRNAs than milk exosomes. Such powerful protective properties limit miRNA breakdown during gastrointestinal transit, promote transepithelial absorption, and support systemic delivery of intact miRNAs to distal tissues following oral administration^[70]^. Collectively, these mechanisms account for the favorable stability of medicinal plant-derived miRNAs within the digestive tract and circulatory system.

In summary, the terminal chemical modifications, CG content characteristics, spatial secondary structures, transport pathways of plant miRNAs, and the protective effects of vesicles collectively form the structural basis for their stable persistence *in vivo*, allowing them to overcome the limitations of conventional nucleic acid degradation mechanisms and maintain biological activity within the digestive tract and circulatory system.

#### Cross-regulation of medicinal plant-derived miRNAs

In recent years, the cross-kingdom regulation of miRNAs has become a hot topic among researchers. As a class of broadly functional molecular regulators, exogenous plant-derived miRNAs not only regulate the expression of their own genes, but also can enter mammalian cells via dietary intake and modulate the expression of specific genes or biological processes associated with human disease therapy.

Extensive experiments have shown that medicinal plants contain miRNAs that share a high degree of homology with human miRNAs. For instance, the 21-nucleotide aba-miRNA-9497 extracted from *Atropa belladonna* exhibits 76% homology with the 21-nucleotide human brain hsa-miRNA-378^[72]^. Although still under debate, some evidence supports that such plant-derived miRNAs may enter the systemic circulation via oral administration and the digestive tract, thereby exerting biological effects^[9]^. Both in vitro and *in vivo* experiments have also confirmed that heat-stable miRNAs extracted from medicinal plants exhibit superior therapeutic activity^[69]^. Distinct from the molecular mechanisms by which natural compounds typically regulate disease pathways, miRNAs can directly modulate the expression of target mRNAs in the human body. This occurs through specific complementary binding to the 3’-untranslated region (UTR) of the targeted mRNA^[73]^, guiding the RNA-induced silencing complex (RISC, a protein complex composed of AGO protein, Dicer protein, and other components) to bind to the mRNA, thereby cutting and degrading it. This leads to decreased expression of the target gene or translational inhibition, ultimately reducing target protein expression and regulating pathophysiological activities. This provides a new direction for research on active substances in medicinal plants^[74]^.

Typically, in animal organisms, a single miRNA can regulate multiple target mRNAs, while a single mRNA may also be co-regulated by multiple miRNAs. In contrast to animal miRNAs, most plant miRNAs require a higher degree of sequence matching with their target mRNAs for binding^[19]^. Recent studies have revealed that some plant miRNAs can be selectively packaged into vesicles, which supports their stability during interspecies transport. Upon entering the human body, they are not degraded but instead transported to target sites where they bind to specific mRNAs, thereby regulating target genes and cellular functions. For example, plant miR-168a specifically targets and binds to the mRNA of low-density lipoprotein receptor-associated protein 1 (LDLRAP1), suppressing its expression in mice and thereby cross-species regulating lipid metabolism^[9]^. MiR-2911 from honeysuckle (*Lonicera japonica*) can be absorbed by the mouse gastrointestinal tract and directly target influenza A virus in the mouse body, inhibiting the expression of polymerase basic protein 2 (PB2) and non-structural protein 1 (NS1) proteins encoded by the virus, thereby suppressing viral replication^[60]^. Sal-miR-1 and Sal-miR-3 derived from *Salvia miltiorrhiza* target and regulate the expression of the OTU deubiquitinase 7B (OTUD7B)/Krüppel-like factor 4 (KLF4)/non-muscle myosin heavy chain IIA (NMHC IIA) axis in the *OTUD7B* gene of both mice and humans, thereby inhibiting vascular remodeling^[75]^. Prunus mume derived extracellular vesicle-like particles (PM-EVLPS) inhibit NIMA-related kinase 7 (NEK7)-NOD-like receptor protein 3 (NLRP3) interaction by delivering active miR-159, blocking NLRP3/apoptosis-associated speck-like protein containing a caspase recruitment domain (ASC)/pro-caspase-1 complex assembly^[76]^, and thereby ameliorating experimental colitis (the underlying mechanism is illustrated in Figure 3). These studies collectively demonstrate the targeted regulatory effects of plant miRNAs [Table 4]. Notably, although accumulating evidence has demonstrated that numerous plant miRNAs can exert biological functions in humans^[82]^, not all plant miRNAs are able to be taken up and utilized by animals. The mechanisms underlying the selective uptake and utilization of plant miRNAs by animals remain to be fully elucidated^[83]^. 

**Figure 3:**
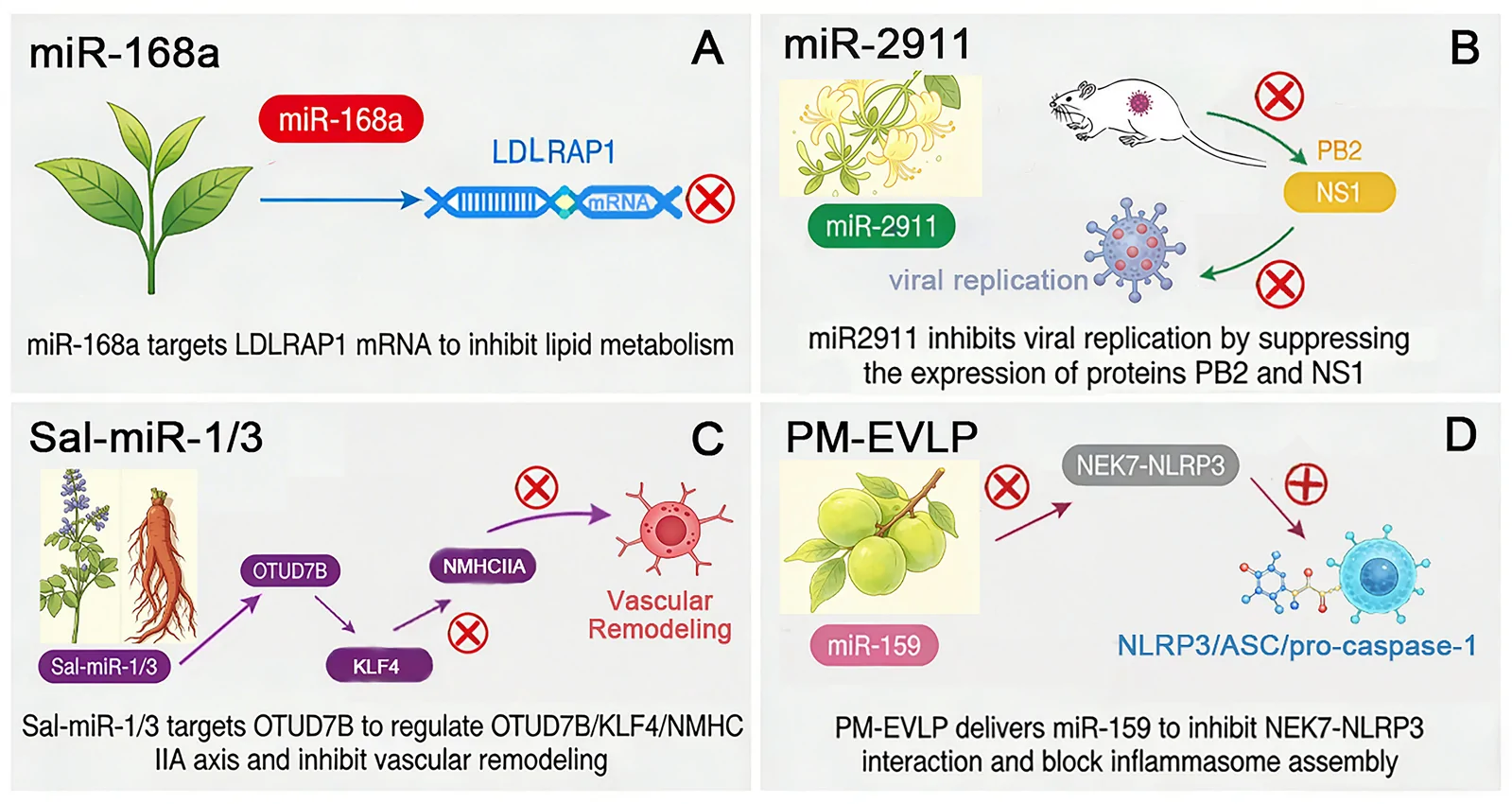
Cross-kingdom regulatory mechanisms of typical medicinal plant-derived miRNAs. (A) Rice-derived miR-168a targets LDLRAP1 mRNA to suppress lipid metabolism; (B) Honeysuckle miR-2911 inhibits viral replication by repressing viral PB2 and NS1 protein expression *in vivo*; (C) Salvia miltiorrhiza Sal-miR-1/3 targets OTUD7B to modulate the OTUD7B/KLF4/NMHC IIA axis and inhibit pathological vascular remodelling; (D) PM-EVLP deliver miR-159 to disrupt NEK7-NLRP3 interaction and block NLRP3 inflammasome assembly. LDLRAP1: Low-density lipoprotein receptor-associated protein 1; miRNA: microRNA; PB2: polymerase basic protein 2; NS1: non-structural protein 1; Sal-miR-1/3: Salvia miltiorrhiza-derived microRNA 1/3; OTUD7B: OTU deubiquitinase 7B; KLF4: Krüppel-like factor 4; NMHC IIA: non-muscle myosin heavy chain IIA; PM-EVLP: plant-derived extracellular vesicle-like particles; NEK7: NIMA-related kinase 7; NLRP3: NOD-like receptor protein 3; ASC: apoptosis-associated speck-like protein containing a caspase recruitment domain; pro-caspase-1: pro-cysteinyl aspartate-specific protease 1.

**Table 4:** The efficacy and mechanism of action of exogenous plant-derived miRNAs

**Efficacy**	**Source**	**Sequence**	**Model/dose**	**Mechanism**	**Ref.**
Antiviral	Honeysuckle	miR-2911	HEK293T/10.5 pmol	miR-2911 can inhibit SARS-CoV-2 replication and accelerate the negative conversion of infected patients	[13]
Protect cardiovascular health	*Salvia miltiorrhiza* Bge.	Sal-miR-1, Sal-miR-3	VSMC, HEK293T, THP-1/50 nM; C57BL/6J mice/10 mg/kg	Sal-miR-1 and Sal-miR-3 counteract thrombin-driven *OTUD7B* upregulation by binding to distinct sites within the *OTUD7B* 3’UTR and attenuate *KLF4* protein abundance through suppressing its deubiquitylation. Reduced *KLF4* alleviates transcriptional repression of the *NMHC IIA* gene, thereby increasing *NMHC IIA* expression. This event represses VSMC migration and monocyte-VSMC adhesion by sustaining the VSMC contractile phenotype	[75]
Regulate lipid metabolism	Tea leaves	miR-21-5p, miR-17-3p, miR-107	HepG-2/100 µg/mL	These miRNAs interact with the corresponding exon of the target gene mRNA in the liver (LDLRAP1) and inhibit the translation of its protein, thereby affecting lipid metabolism and reducing the clearance rate of low-density lipoprotein in plasma	[77]
Microbial regulation	Tomato	miR1001	*Botrytis cinerea*/10 µM	Downregulation of the *Bcin03g02170.1* gene encoding an ATP-dependent metallopeptidase and the *Bcin10g01400.1* gene encoding a cysteine endopeptidase in *Botrytis cinerea* inhibits conidial germination and pathogenicity, thereby preventing self-infection by the fungus	[78]
Anti-inflammatory	Ginger	Osa-miR-164d	RAW264.7 macrophages/40 and 80 µg/mL; C57BL/6 mice/200 µg/day	Targets three key inflammation-related genes on a pathway activated by nuclear factors in B cells, thereby participating in important physiological processes such as immune and inflammatory responses in animals	[79]
Anti-fibrosis	*Rhodiola crenulata*	HJT-sRNA-m7	MRC-5, A549, NCI-N87, HEK293T; C57BL/6J mice/250 µg/kg	Targeting key proteins involved in the development and progression of pulmonary fibrosis: α-SMA, fibronectin, and COL3A1	[80]
Regulate gut microbiota	Rehmanniae Radix	miR-7972	BALB/c mice/200 nmol	miR-7972 downregulated the expression of *GPR161*, activating the Hedgehog pathway, and inhibited the biofilm formation of *Escherichia coli* via targeting the virulence gene sxt2	[81]

miRNA: MicroRNA; SARS-CoV-2: severe acute respiratory syndrome coronavirus 2; VSMC: vascular smooth muscle cell; mRNA: messenger RNA; LDLRAP1: low-density lipoprotein receptor-associated protein 1; UTR: untranslated region; OTUD7B: OTU deubiquitinase 7B; KLF4: Krüppel-like factor 4; NMHC IIA: non-muscle myosin heavy chain IIA; ATP: adenosine triphosphate; α-SMA: alpha-smooth muscle actin; COL3A1: collagen type III alpha 1; GPR161: G protein-coupled receptor 161; sRNA: small non-coding RNA.

## PHARMACEUTICAL POTENTIAL AND CHALLENGES OF NUCLEIC ACIDS IN MEDICINAL PLANTS

miRNAs are critical regulatory nucleic acids controlling extensive gene expression networks in organisms. Combined comparative genomic analysis revealed that more than 60% of human protein-coding genes harbor miRNA binding sites maintained by purifying selection^[21]^, making miRNA-based therapeutics an attractive research hotspot. To date, multiple types of approved synthetic nucleic acid therapeutics, such as antisense oligonucleotides (ASOs), siRNAs, and mRNA vaccines, have validated the druggability of nucleic acid molecules [Supplementary Table 1]^[84]^. Meanwhile, a series of synthetic miRNA-targeted oligonucleotides have advanced into clinical trials, including miRNA mimics and anti-miR inhibitors [Table 5].

**Table 5:** Selected miRNA drugs entering clinical trials

**Drugs**	**Target**	**Disease**	**Stage of development**	**Ref.**
Miravirsen	miR-122	HCV	Phase 2 study	[85]
TargomiRs (MesomiR-1)	miR-16	Malignant pleural mesothelioma	Phase 1 study	[86]
MRX34	miR-34a	Advanced solid tumors	Phase 1 study	[87]
Lademirsen	miR-21	Alport syndrome	Phase 2 study	[88]
Cobomarsen (MRG-106)	miR-155	CTCL/mycosis fungoides	Phase 2 study	[89]
AMT-130	miHTT	Huntington’s disease	Phase 2 study	[90]
MRG-110	miR-92a	Cardiovascular disease and wound healing	Phase 1 study	[91]
RGLS4326	miR-17	ADPKD	Phase 1 study	[92]
CDR132L	miR-132	Heart failure	Phase 1b study	[93]

HCV: Hepatitis C virus; miR: microRNA; CTCL: cutaneous T-cell lymphoma; ADPKD: autosomal dominant polycystic kidney disease; miHTT: microRNA targeting huntingtin mRNA.

Two mainstream design strategies are adopted for synthetic miRNA therapeutics: supplementation of miRNAs with active functions via miRNA mimics, and silencing pathogenic miRNAs using synthetic antisense inhibitors^[94]^. Nevertheless, although these candidates exhibit promising effects in preclinical experiments, their clinical translation is hampered by multiple *in vivo* obstacles. Naked synthetic miRNAs are rapidly degraded by nucleases and undergo fast renal clearance, leading to low bioavailability and suboptimal pharmacokinetic profiles^[95-98]^. Widely explored delivery carriers, including lipid nanoparticles, conventional liposomes, and extracellular vesicles, still have inherent limitations such as restricted tissue targeting, circulation instability, and barriers to standardized large-scale manufacture^[99-101]^. Furthermore, off-target effects, potential immunogenicity, and imperfect industrial and clinical evaluation frameworks further restrict their broad clinical application^[102-107]^.

Against this background, naturally occurring nucleic acids derived from medicinal plants represent a promising alternative resource. Growing evidence confirms that plant endogenous miRNAs can achieve cross-kingdom regulation and modulate gene expression within mammalian cells. Distinct from chemically synthesized miRNA oligonucleotides, plant-derived nucleic acids exist within complex herbal matrices. Coexisting phytochemical constituents in medicinal plant extracts may naturally improve nucleic acid stability and facilitate *in vivo* delivery. In addition, plant-originated miRNAs generally possess relatively low immunogenicity and multi-target regulatory characteristics, matching the holistic pharmacological features of herbal medicines.

Even so, the exploitation of nucleic acids from medicinal plants remains in the preliminary stage. Systematic *in vivo* studies are still required to characterize the absorption, distribution and functional persistence of plant miRNAs after administration, verify cross-kingdom bioactivity, and develop suitable preparation strategies. Breaking through these limitations will help unlock the pharmaceutical potential of native nucleic acids in medicinal plants and provide new candidates to complement synthetic miRNA therapeutics.

## CONCLUSION AND FUTURE DIRECTIONS

This review systematically sorts out the classification, tissue distribution, optimized extraction strategies, multi-layered *in vivo* stabilization mechanisms, and cross-kingdom pharmacological regulatory activities of nucleic acids represented by miRNAs derived from medicinal plants. Whereas long-standing research has focused on herbal secondary metabolites such as flavonoids and terpenoids, plant-endogenous miRNAs have emerged as a novel class of orally bioactive natural components that may mediate interspecies gene regulation in mammals. Multiple intrinsic structural features and extrinsic transport pathways jointly maintain their biological activity amid harsh gastrointestinal and circulatory environments, including terminal 2’-O-methylation, high CG base composition, stable hairpin secondary conformations, low-pH-dependent SIDT1-mediated gastric epithelial absorption, and lipid encapsulation within plant exosome-like nanovesicles.

Cumulative experiments have validated the broad-spectrum therapeutic potential of medicinal plant miRNAs covering antitumor, lipid metabolism regulation, antiviral, anti-inflammatory, and cardiovascular protective effects. Currently, two core development frameworks dominate synthetic miRNA therapeutics: supplementing tumor-suppressive signals via chemically modified miRNA mimics, and silencing oncogenic transcripts through synthetic complementary miRNA inhibitors. Despite their preclinical efficacy, synthetic nucleic acid drugs face prominent translational obstacles, including rapid nuclease degradation, low oral bioavailability, off-target interference, and immunogenic risks. In contrast, medicinal plant miRNAs possess unique translational advantages after thousands of years of oral screening in traditional Asian herbal therapy, featuring superior biocompatibility, low adverse reaction rates, and minimal immunogenicity relative to artificially synthesized RNA agents. Nevertheless, prominent research bottlenecks still restrict their clinical translation: synergistic regulatory networks between plant miRNAs and coexisting herbal secondary metabolites remain insufficiently clarified, and standardized oral delivery carriers that sustain miRNA stability during* in vivo* circulation have not been fully established.

Building on the current research gaps summarized above, future work should prioritize several interconnected research orientations to advance the translational development of medicinal plant-derived miRNA therapeutics. First, multi-omics analytical systems combining transcriptomics, proteomics, and metabolomics should be deployed to systematically decode the synergistic cross-regulatory relationships between plant miRNAs and herbal phytochemicals, while rigorous *in vivo* trials are required to resolve ongoing academic disputes regarding the oral bioavailability, intestinal absorption efficiency, and tissue distribution characteristics of dietary plant miRNAs. Second, species-specific extraction workflows tailored to diverse medicinal herbs and traditional decoction processing modes need to be standardized to eliminate interference from polyphenols and polysaccharides and improve the yield and integrity of recovered miRNAs; drawing on the outstanding protective performance of natural nanovesicles, low-cost natural vesicle delivery platforms should be developed to strengthen miRNA tolerance to digestive stress. Third, scalable isolation, surface modification, and tissue-targeted transformation technologies for plant-derived exosome-like nanovesicles should be explored, and quantitative pharmacokinetic assessments of encapsulated plant miRNAs need to be performed in mammalian models to characterize their intestinal uptake and systemic metabolic profiles. Fourth, a complete preclinical safety evaluation system dedicated to oral plant miRNA preparations must be constructed to systematically assess acute and chronic toxicity, immunogenicity, and long-term off-target gene interference; unified quality control indicators, mass production specifications, and clinical trial evaluation criteria also need to be formulated to support the industrialization of natural plant nucleic acid drugs as complementary alternatives to synthetic RNA therapeutics. Additionally, high-throughput small RNA sequencing and degradome analysis should be applied to excavate novel functional miRNAs from underexplored medicinal plant varieties, enriching the resource library of natural regulatory nucleic acids for new drug screening and development.

## DECLARATIONS

### AI and AI-assisted tools statement

During the preparation of this manuscript, the AI tool DeepSeek (version 1.7.11, released 2026-03-26) was used solely for language polishing. In addition, the AI tool Doubao (version Doubao-Seed-2.1 Pro, released 2026-06-23) was used to generate individual visual elements for the graphical abstract and figures, which were subsequently assembled and edited by the authors using Adobe Photoshop 2024. These tools did not influence the study design, data collection, analysis, interpretation, or scientific content of the work. All authors take full responsibility for the accuracy, integrity, and final content of the manuscript.

### Copyright

© The Author(s) 2026.

## References

[1] Yang L, Yang Y, Huang L, Cui X, Liu Y (2023). From single- to multi-omics: future research trends in medicinal plants. Brief Bioinform.

[2] M S, Puthiyedath R, Pillai ZS (2025). Drug-herb interactions: a challenge and clinical concern in primary healthcare. Front Med.

[3] Zhou W, Liu H, Qiu LZ (2021). Cardiac efficacy and toxicity of aconitine: a new frontier for the ancient poison. Med Res Rev.

[4] Ren Y, Qu S (2023). Constituent isoflavones of *Puerariae radix* as a potential neuroprotector in cognitive impairment: evidence from preclinical studies. Ageing Res Rev.

[5] Zhang H, Xiong J, Wang Q (2025). Chrysophanol delays aging via insulin/IGF-1 signaling pathway. Free Radic Biol Med.

[6] Yang HS, Mauki DH, Zheng YX, Wang TH, He XY (2025). Terpenoids: a promising traditional chinese medicine for neuropathic pain relief. Pharmacol Res.

[7] Chai M, Gao B, Wang S (2025). Leveraging plant-derived nanovesicles for advanced nucleic acid-based gene therapy. Theranostics.

[8] Fire A, Xu S, Montgomery MK, Kostas SA, Driver SE, Mello CC (1998). Potent and specific genetic interference by double-stranded RNA in *Caenorhabditis elegans*. Nature.

[9] Zhang L, Hou D, Chen X (2012). Exogenous plant MIR168a specifically targets mammalian LDLRAP1: evidence of cross-kingdom regulation by microRNA. Cell Res.

[10] Huang F, Du J, Liang Z (2019). Large-scale analysis of small RNAs derived from traditional Chinese herbs in human tissues. Sci China Life Sci.

[11] Shi R, Tan W, Jin H (2025). MicroRNA-enriched plant-derived exosomes alleviate colitis by modulating systemic immunity, metabolic homeostasis, and gut microbiota. Adv Sci.

[12] Liu J, Wang F, Song H (2021). Soybean-derived gma-miR159a alleviates colon tumorigenesis by suppressing TCF7/MYC in mice. J Nutr Biochem.

[13] Zhou LK, Zhou Z, Jiang XM (2020). Absorbed plant MIR2911 in honeysuckle decoction inhibits SARS-CoV-2 replication and accelerates the negative conversion of infected patients. Cell Discov.

[14] Binder AK, Bremm F, Dörrie J, Schaft N (2024). Non-coding RNA in tumor cells and tumor-associated myeloid cells-function and therapeutic potential. Int J Mol Sci.

[15] Zhan J, Meyers BC (2023). Plant small RNAs: their biogenesis, regulatory roles, and functions. Annu Rev Plant Biol.

[16] Yang HH, Zheng BL (2025). Biogenesis, action, function of plant small RNAs and their potential application in agriculture. Yi Chuan.

[17] Tang Q, Khvorova A (2024). RNAi-based drug design: considerations and future directions. Nat Rev Drug Discov.

[18] Zhang Z, Yu M, Hu G, Gan Y (2025). The new era of siRNA therapy: advances in cancer treatment. Clin Transl Med.

[19] Ajila V, Colley L, Ste-Croix DT (2023). P-TarPmiR accurately predicts plant-specific miRNA targets. Sci Rep.

[20] Yang L, Feng H (2023). Cross-kingdom regulation by plant-derived miRNAs in mammalian systems. Animal Model Exp Med.

[21] Diener C, Keller A, Meese E (2024). The miRNA-target interactions: an underestimated intricacy. Nucleic Acids Res.

[22] Lewis BP, Burge CB, Bartel DP (2005). Conserved seed pairing, often flanked by adenosines, indicates that thousands of human genes are microRNA targets. Cell.

[23] Babak T, Zhang W, Morris Q, Blencowe BJ, Hughes TR (2004). Probing microRNAs with microarrays: tissue specificity and functional inference. RNA.

[24] Lee RC, Feinbaum RL, Ambros V (1993). The C. elegans heterochronic gene lin-4 encodes small RNAs with antisense complementarity to lin-14. Cell.

[25] Yang Y, Chen X, Chen J, Xu H, Li J, Zhang Z (2011). Differential miRNA expression in Rehmannia glutinosa plants subjected to continuous cropping. BMC Plant Biol.

[26] Xu T, Wang B, Liu X, Feng R, Dong M, Chen J (2012). Microarray-based identification of conserved microRNAs from Pinellia ternata. Gene.

[27] Dong M, Yang D, Lang Q, Zhou W, Xu S, Xu T (2013). Microarray and degradome sequencing reveal microRNA differential expression profiles and their targets in Pinellia pedatisecta. PLoS One.

[28] Fan R, Li Y, Li C, Zhang Y (2015). Differential microRNA analysis of glandular trichomes and young leaves in *Xanthium strumarium* L. reveals their putative roles in regulating terpenoid biosynthesis. PLoS One.

[29] Wei R, Qiu D, Wilson IW (2015). Identification of novel and conserved microRNAs in Panax notoginseng roots by high-throughput sequencing. BMC Genomics.

[30] Gao ZH, Wei JH, Yang Y, Zhang Z, Xiong HY, Zhao WT (2012). Identification of conserved and novel microRNAs in Aquilaria sinensis based on small RNA sequencing and transcriptome sequence data. Gene.

[31] Jung I, Kang H, Kim JU, Chang H, Kim S, Jung W (2018). The mRNA and miRNA transcriptomic landscape of Panax ginseng under the high ambient temperature. BMC Syst Biol.

[32] Gu L, Lai Y, Zhang G (2024). Genome-wide identification of the *Rehmannia glutinosa* miRNA family and exploration of their expression characteristics caused by the replant disease formation-related principal factor. Genes.

[33] He X, Shenkute AG, Wang W, Xu S (2018). Characterization of conserved and novel microRNAs in *Lilium lancifolium* Thunb. by high-throughput sequencing. Sci Rep.

[34] Nadiya F, Anjali N, Thomas J, Gangaprasad A, Sabu KK (2019). Deep sequencing identified potential miRNAs involved in defence response, stress and plant growth characteristics of wild genotypes of cardamom. Plant Biol.

[35] Boke H, Ozhuner E, Turktas M, Parmaksiz I, Ozcan S, Unver T (2015). Regulation of the alkaloid biosynthesis by miRNA in opium poppy. Plant Biotechnol J.

[36] Hao DC, Yang L, Xiao PG, Liu M (2012). Identification of Taxus microRNAs and their targets with high-throughput sequencing and degradome analysis. Physiol Plant.

[37] Shao J, Wang L, Liu Y (2020). Identification of milRNAs and their target genes in Ganoderma lucidum by high-throughput sequencing and degradome analysis. Fungal Genet Biol.

[38] Wang Y, Chen Y, Peng M (2023). Identification of microRNA and analysis of target genes in Panax ginseng. Chin Herb Med.

[39] Zhou H, Jiang M, Li J, Xu Y, Li C, Lu S (2024). Genome-wide identification and functional analysis of *Salvia miltiorrhiza* microRNAs reveal the negative regulatory role of Smi-miR159a in phenolic acid biosynthesis. Int J Mol Sci.

[40] Xia H, Zhang L, Wu G (2016). Genome-wide identification and characterization of microRNAs and target genes in lonicera japonica. PLoS One.

[41] Sasi S, Krishnan S, Kodackattumannil P (2023). DNA-free high-quality RNA extraction from 39 difficult-to-extract plant species (representing seasonal tissues and tissue types) of 32 families, and its validation for downstream molecular applications. Plant Methods.

[42] Brown GN (1969). Changes in ribonucleic acid fractions during maturation of mimosa epicotyl tissues. Plant Physiol.

[43] Cho Y, Fernandes J, Kim SH, Walbot V (2002). Gene-expression profile comparisons distinguish seven organs of maize. Genome Biol.

[44] Bélanger S, Zhan J, Yu Y, Meyers BC (2024). Comparative RNA profiling identifies stage-specific phasiRNAs and coexpressed *Argonaute *genes in Bambusoideae and Pooideae species. Plant Cell.

[45] Kaushik N, Noorani MS, Shukla K (2024). CTAB protocol for obtaining high-quality total RNA from *Hibiscus rosa*-sinensis and other related plants of the family malvaceae. ACS Omega.

[46] Peng J, Xia Z, Chen L (2014). Rapid and efficient isolation of high-quality small RNAs from recalcitrant plant species rich in polyphenols and polysaccharides. PLoS One.

[47] Yang BH, Liu BS, Chen ZL (2021). DNA extraction with TRIzol reagent using a silica column. Anal Sci.

[48] (2010). Rio DC, Ares M Jr, Hannon GJ, Nilsen TW. Purification of RNA by SDS solubilization and phenol extraction. Cold Spring Harb Protoc.

[49] Vennapusa AR, Somayanda IM, Doherty CJ, Jagadish SVK (2020). A universal method for high-quality RNA extraction from plant tissues rich in starch, proteins and fiber. Sci Rep.

[50] Wang L, Stegemann JP (2010). Extraction of high quality RNA from polysaccharide matrices using cetyltrimethylammonium bromide. Biomaterials.

[51] Yang Z, He CL, Li FF

[52] Pei Y, Mamtimin T, Ji J (2021). The guanidine thiocyanate-high EDTA method for total microbial RNA extraction from severely heavy metal-contaminated soils. Microb Biotechnol.

[53] Baelde JJ, Bergijk EC, Hoedemaeker PJ, de Heer E, Bruijn JA (1994). Optimal method for RNA extraction from mouse glomeruli. Nephrol Dial Transplant.

[54] Shi R, Lewis RS, Panthee DR (2018). Filter paper-based spin column method for cost-efficient DNA or RNA purification. PLoS One.

[55] Wang Y, Peng M, Wang W (2019). Verification of miRNAs in ginseng decoction by high-throughput sequencing and quantitative real-time PCR. Heliyon.

[56] Luo Y, Wang P, Wang X (2017). Detection of dietetically absorbed maize-derived microRNAs in pigs. Sci Rep.

[57] Xie W, Melzig MF (2018). The stability of medicinal plant microRNAs in the herb preparation process. Molecules.

[58] Wang X, Ren X, Ning L, Wang P, Xu K (2020). Stability and absorption mechanism of typical plant miRNAs in an* in vitro* gastrointestinal environment: Basis for their cross-kingdom nutritional effects. J Nutr Biochem.

[59] Díez-Sainz E, Milagro FI, Aranaz P, Riezu-Boj JI, Lorente-Cebrián S (2024). MicroRNAs from edible plants reach the human gastrointestinal tract and may act as potential regulators of gene expression. J Physiol Biochem.

[60] Zhou Z, Li X, Liu J (2015). Honeysuckle-encoded atypical microRNA2911 directly targets influenza A viruses. Cell Res.

[61] Cavallini A, Minervini F, Garbetta A (2018). High degradation and no bioavailability of artichoke miRNAs assessed using an in vitro digestion/Caco-2 cell model. Nutr Res.

[62] Qin X, Wang X, Xu K (2022). Digestion of plant dietary miRNAs starts in the mouth under the protection of coingested food components and plant-derived exosome-like nanoparticles. J Agric Food Chem.

[63] Del Pozo-Acebo L, López de Las Hazas MC, Margollés A, Dávalos A, García-Ruiz A (2021). Eating microRNAs: pharmacological opportunities for cross-kingdom regulation and implications in host gene and gut microbiota modulation. Br J Pharmacol.

[64] Li J, Yang Z, Yu B, Liu J, Chen X (2005). Methylation protects miRNAs and siRNAs from a 3’-end uridylation activity in Arabidopsis. Curr Biol.

[65] Xu Q, Wang J, Zhang Y (2024). Atypical plant miRNA cal-miR2911: robust stability against food digestion and specific promoting effect on *Bifidobacterium *in mice. J Agric Food Chem.

[66] Philip A, Ferro VA, Tate RJ (2015). Determination of the potential bioavailability of plant microRNAs using a simulated human digestion process. Mol Nutr Food Res.

[67] Chen Q, Zhang F, Dong L (2021). SIDT1-dependent absorption in the stomach mediates host uptake of dietary and orally administered microRNAs. Cell Res.

[68] Sun CR, Xu D, Yang F (2024). Human SIDT1 mediates dsRNA uptake via its phospholipase activity. Cell Res.

[69] Zhao Y, Mo B, Chen X (2012). Mechanisms that impact microRNA stability in plants. RNA Biol.

[70] Umezu T, Takanashi M, Murakami Y (2021). Acerola exosome-like nanovesicles to systemically deliver nucleic acid medicine via oral administration. Mol Ther Methods Clin Dev.

[71] Tembo KM, Wang X, Bolideei M (2025). Exploring the bioactivity of microRNAs originated from plant-derived exosome-like nanoparticles (PELNs): current perspectives. J Nanobiotechnology.

[72] Avsar B, Zhao Y, Li W, Lukiw WJ (2020). *Atropa belladonna *expresses a microRNA (*aba*-*miRNA*-9497) highly homologous to *Homo sapiens* miRNA-378 (*hsa*-*miRNA*-378); both miRNAs target the 3’-Untranslated Region (3’-UTR) of the mRNA encoding the neurologically relevant, zinc-finger transcription factor ZNF-691. Cell Mol Neurobiol.

[73] Bartel DP (2009). MicroRNAs: target recognition and regulatory functions. Cell.

[74] Sun M, Xu S, Mei Y (2022). MicroRNAs in medicinal plants. Int J Mol Sci.

[75] Yang GS, Zheng B, Qin Y (2020). * Salvia miltiorrhiza*-derived miRNAs suppress vascular remodeling through regulating OTUD7B/KLF4/NMHC IIA axis. Theranostics.

[76] Lv Q, Yang H, Xie Y (2025). Prunus mume derived extracellular vesicle-like particles alleviate experimental colitis via disrupting NEK7-NLRP3 interaction and inflammasome activation. J Nanobiotechnology.

[77] Lei X, Li H, Chen S (2025). Tea leaf exosome-like nanoparticles (TELNs) improve oleic acid-induced lipid metabolism by regulating miRNAs in HepG-2 cells. Bioresour Bioprocess.

[78] Meng X, Jin W, Wu F (2020). Novel tomato miRNA miR1001 initiates cross-species regulation to suppress the conidiospore germination and infection virulence of *Botrytis cinerea* in vitro. Gene.

[79] Yan L, Cao Y, Hou L (2025). Ginger exosome-like nanoparticle-derived miRNA therapeutics: a strategic inhibitor of intestinal inflammation. J Adv Res.

[80] Du J, Liang Z, Xu J (2019). Plant-derived phosphocholine facilitates cellular uptake of anti-pulmonary fibrotic HJT-sRNA-m7. Sci China Life Sci.

[81] Qiu FS, Wang JF, Guo MY (2023). Rgl-exomiR-7972, a novel plant exosomal microRNA derived from fresh Rehmanniae Radix, ameliorated lipopolysaccharide-induced acute lung injury and gut dysbiosis. Biomed Pharmacother.

[82] Masood M, Everett CP, Chan SY, Snow JW (2016). Negligible uptake and transfer of diet-derived pollen microRNAs in adult honey bees. RNA Biol.

[83] Shi L, Guo C, Fang M, Yang Y, Yin F, Shen Y (2024). Cross-kingdom regulation of plant microRNAs: potential application in crop improvement and human disease therapeutics. Front Plant Sci.

[84] Miao Y, Fu C, Yu Z (2024). Current status and trends in small nucleic acid drug development: leading the future. Acta Pharm Sin B.

[85] van der Ree MH, van der Meer AJ, de Bruijne J (2014). Long-term safety and efficacy of microRNA-targeted therapy in chronic hepatitis C patients. Antiviral Res.

[86] van Zandwijk N, Pavlakis N, Kao SC (2017). Safety and activity of microRNA-loaded minicells in patients with recurrent malignant pleural mesothelioma: a first-in-man, phase 1, open-label, dose-escalation study. Lancet Oncol.

[87] Hong DS, Kang YK, Borad M (2020). Phase 1 study of MRX34, a liposomal miR-34a mimic, in patients with advanced solid tumours. Br J Cancer.

[88] Gale DP, Gross O, Wang F (2024). ; HERA Clinical Trial Group. A randomized controlled clinical trial testing effects of lademirsen on kidney function decline in adults with alport syndrome. Clin J Am Soc Nephrol.

[89] Seto AG, Beatty X, Lynch JM (2018). Cobomarsen, an oligonucleotide inhibitor of miR-155, co-ordinately regulates multiple survival pathways to reduce cellular proliferation and survival in cutaneous T-cell lymphoma. Br J Haematol.

[90] Farag M, Tabrizi SJ, Wild EJ (2025). Huntington’s disease clinical trials update: March 2025. J Huntingtons Dis.

[91] Abplanalp WT, Fischer A, John D (2020). Efficiency and target derepression of anti-miR-92a: results of a first in human study. Nucleic Acid Ther.

[92] Owen T, Cole I, Drygin D (2026). Pharmacokinetics, safety, and tolerability of anti-miR-17 oligonucleotide RGLS4326 in healthy adult subjects. J Clin Pharmacol.

[93] Täubel J, Hauke W, Rump S (2021). Novel antisense therapy targeting microRNA-132 in patients with heart failure: results of a first-in-human Phase 1b randomized, double-blind, placebo-controlled study. Eur Heart J.

[94] Di Martino MT, Tagliaferri P, Tassone P (2025). MicroRNA in cancer therapy: breakthroughs and challenges in early clinical applications. J Exp Clin Cancer Res.

[95] Wang Z, Peng Y, Zhou H, Zhang M, Ju D, Chen Z (2025). MiRNA-based drugs: challenges and delivery strategies. Appl Microbiol Biotechnol.

[96] Sharma P, Kumar A, Agarwal T (2022). Nucleic acid-based therapeutics for dermal wound healing. Int J Biol Macromol.

[97] Zare M, Pemmada R, Madhavan M (2022). Encapsulation of miRNA and siRNA into nanomaterials for cancer therapeutics. Pharmaceutics.

[98] Pagoni M, Cava C, Sideris DC (2023). miRNA-based technologies in cancer therapy. J Pers Med.

[99] Traber GM, Yu AM (2023). RNAi-based therapeutics and novel RNA bioengineering technologies. J Pharmacol Exp Ther.

[100] Asakiya C, Zhu L, Yuhan J, Zhu L, Huang K, Xu W (2022). Current progress of miRNA-derivative nucleotide drugs: modifications, delivery systems, applications. Expert Opin Drug Deliv.

[101] Manikkath J, Jishnu PV, Wich PR, Manikkath A, Radhakrishnan R (2022). Nanoparticulate strategies for the delivery of miRNA mimics and inhibitors in anticancer therapy and its potential utility in oral submucous fibrosis. Nanomedicine.

[102] Zhang Y, Wang Z, Gemeinhart RA (2013). Progress in microRNA delivery. J Control Release.

[103] Seyhan AA (2024). Trials and tribulations of microRNA therapeutics. Int J Mol Sci.

[104] Diener C, Keller A, Meese E (2022). Emerging concepts of miRNA therapeutics: from cells to clinic. Trends Genet.

[105] Sainz V, Conniot J, Matos AI (2015). Regulatory aspects on nanomedicines. Biochem Biophys Res Commun.

[106] Reda El Sayed S, Cristante J, Guyon L, Denis J, Chabre O, Cherradi N (2021). MicroRNA therapeutics in cancer: current advances and challenges. Cancers.

[107] Ren T, Ma L, Fu P, Zhang C (2025). Nanoparticles used for the delivery of RNAi-based therapeutics. Pharmaceutics.

