# Supplementary material for: Nucleic acids in medicinal plants and their pharmaceutical potential
Source: Extracell Vesicles Circ Nucl Acids. 2026 Sep 18;7(3):1517–33. doi: 10.20517/evcna.2026.57 (PMC13673453; doi:10.20517/evcna.2026.57)
Supplement: Supplementary file 1 [file evcna-7-3-1517-SupplementaryMaterials.pdf]

Supplementary Materials

Nucleic acids in medicinal plants and their pharmaceutical potential

Xiaoyan Qin[1], Fangwen Jiao[2], Hongzhi Qiao[1,3]

[1]Jiangsu Key Laboratory of Medicinal Substance and Utilization of Fresh Chinese Medicine, School of Pharmacy, Nanjing University of Chinese Medicine, Nanjing 210023, Jiangsu, China.

[2]School of Medicine, Nanjing University of Chinese Medicine, Nanjing 210023, Jiangsu, China.

[3]Jiangsu Engineering Research Center for Efficient Delivery System of traditional Chinese medicine (TCM), School of Pharmacy, Nanjing University of Chinese Medicine, Nanjing 210023, Jiangsu, China.

Correspondence to: Dr. Fangwen Jiao, School of Medicine, Nanjing University of Chinese Medicine, Nanjing 210023, Jiangsu, China. E-mail: fangwen@njucm.edu.cn; Dr. Hongzhi Qiao, Jiangsu Engineering Research Center for Efficient Delivery System of traditional Chinese medicine (TCM), School of Pharmacy, Nanjing University of Chinese Medicine, Nanjing 210023, Jiangsu, China. E-mail: qiaohz@njucm.edu.cn

Supplementary Table 1. Globally marketed small nucleic acid medicines

Name	Drug category	Indications	Listing date	Ref.
Fomivirsen	ASO	Cytomegalovirus (CMV)	1998	[1]
Mipomersen	ASO	Homozygous Familial Hypercholesterolaemia (HoFH)	2013	[2]
Eteplirsen	ASO	Duchenne Muscular Dystrophy (DMD)	2016	[3]
Nusinersen	ASO	Spinal Muscular Atrophy (SMA)	2016	[4]
Inotersen	ASO	Mutant and Wild-type Human Transthyretin (TTR)	2018	[5]
Volanesorsen	ASO	Familial Chylomicronemia Syndrome (FCS)	2019	[6]
Golodirsen	ASO	Duchenne Muscular Dystrophy (DMD)	2019	[7]
Viltolarsen	ASO	Duchenne Muscular Dystrophy (DMD)	2020	[8]
Casimersen	ASO	Duchenne Muscular Dystrophy (DMD)	2021	[9]
Tofersen	ASO	Amyotrophic Lateral Sclerosis (ALS)	2023	[10]
Eplontersen	ASO	TTR-mediated Amyloidosis (ATTR)	2023	[11]
Imetelstat	ASO	Myelodysplastic Syndromes (MDS)	2024	[12]
Olezarsen	ASO	Familial Chylomicronaemia Syndrome (FCS) and Severe Hypertriglyceridaemia	2024	[13]
Donidalorsen	ASO	Hereditary Angioedema (HAE)	2025	[14]
Patisiran	siRNA	Polyneuropathy of Hereditary TTR-Mediated Amyloidosis (hATTR)	2018	[15]
Givosiran	siRNA	Acute Hepatic Porphyria (AHP)	2019	[16]

Lumasiran	siRNA	Primary Hyperoxaluria Type 1 (PH1)	2020	[17]
Inclisiran	siRNA	Primary Hypercholesterolaemia	2020	[18]
Vutrisiran	siRNA	Amyloid Transthyretin-mediated (ATTR) Amyloidosis	2022	[19]
Nedosiran	siRNA	Primary Hyperoxaluria (PH)	2023	[20]
Fitusiran	siRNA	Haemophilia A and B with or without Inhibitors	2025	[21]
Plozasiran		Familial Chylomicronemia Syndrome (FCS)	2025	[22]
Pegaptanib	Aptamer	Neovascular Age-related Macular Degeneration	2004	[23]
Avacincaptad pegol	Aptamer	Geographic Atrophy Secondary to Age-related Macular Degeneration	2023	[24]

Reference

1. Marwick C. First "antisense" drug will treat CMV retinitis.*Jama.* 1998;280:871. [PMID: 9739955 DOI: 10.1001/jama.280.10.871-JMN0909-6-1]

2. Hair P, Cameron F, Mckeage K. Mipomersen sodium: first global approval.*Drugs.* 2013;73:487–493. [PMID: 23564617 DOI: 10.1007/s40265-013-0042-2]

3. Syed Y Y. Eteplirsen: First Global Approval.*Drugs.* 2016;76:1699–1704. [PMID: 27807823 DOI: 10.1007/s40265-016-0657-1]

4. Hoy S M. Nusinersen: First Global Approval.*Drugs.* 2017;77:473–479. [PMID: 28229309 DOI: 10.1007/s40265-017-0711-7]

5. Keam S J. Inotersen: First Global Approval.*Drugs.* 2018;78:1371–1376. [PMID: 30120737 DOI: 10.1007/s40265-018-0968-5]

6. Paik J, Duggan S. Volanesorsen: First Global Approval.*Drugs.* 2019;79:1349 – 1354. [PMID: 31301033 DOI: 10.1007/s40265-019-01168-z]

7. Heo Y A. Golodirsen: First Approval.*Drugs.* 2020;80:329 – 333. [PMID: 32026421 DOI: 10.1007/s40265-020-01267-2]

8. Dhillon S. Viltolarsen: First Approval.*Drugs.* 2020;80:1027 – 1031. [PMID: 32519222 DOI: 10.1007/s40265-020-01339-3]

9. Shirley M. Casimersen: First Approval.*Drugs.* 2021;81:875 – 879. [PMID: 33861387 DOI: 10.1007/s40265-021-01512-2]

10. Blair H A. Tofersen: First Approval.*Drugs.* 2023;83:1039 – 1043. [PMID: 37316681 DOI: 10.1007/s40265-023-01904-6]

11. Nie T. Eplontersen: First Approval.*Drugs.* 2024;84:473 – 478. [PMID: 38413492 DOI: 10.1007/s40265-024-02008-5]

12. Keam S J. Imetelstat: First Approval.*Drugs.* 2024;84:1149 – 1155. [PMID: 39162963 DOI: 10.1007/s40265-024-02080-x]

13. Syed Y Y. Olezarsen: First Approval.*Drugs.* 2025;85:571 – 576. [PMID: 40074987 DOI: 10.1007/s40265-025-02166-0]

14. Syed Y Y. Donidalorsen: First Approval.*Drugs.* 2025;86:243-248. [PMID: 41222832 DOI: 10.1007/s40265-025-02257-y]

15. Hoy S M. Patisiran: First Global Approval.*Drugs.* 2018;78:1625–1631. [PMID: 30251172 DOI: 10.1007/s40265-018-0983-6]

16. Scott L J. Givosiran: First Approval.*Drugs.* 2020;80:335 – 339. [PMID: 32034693 DOI: 10.1007/s40265-020-01269-0]

17. Scott L J, Keam S J. Lumasiran: First Approval.*Drugs.* 2021;81:277–282. [PMID: 33405070 DOI: 10.1007/s40265-020-01463-0]

18. Lamb Y N. Inclisiran: First Approval.*Drugs.* 2021;, (3)389 – 395. [PMID: 33620677 DOI: 10.1007/s40265-021-01473-6]

19. Keam S J. Vutrisiran: First Approval.*Drugs.* 2022;82:1419 – 1425. [PMID: 35997942 DOI: 10.1007/s40265-022-01765-5]

20. Syed Y Y. Nedosiran: First Approval.*Drugs.* 2023;83:1729 – 1733. [PMID: 38060091 DOI: 10.1007/s40265-023-01976-4]

21. Lee A. Fitusiran: First Approval.*Drugs.* 2025;85:1073 – 1077. [PMID: 40579682 DOI: 10.1007/s40265-025-02203-y]

22. Syed Y Y. Plozasiran: First Approval. *Drugs.* 2026. [PMID: 41787196 DOI: 10.1007/s40265-026-02293-2]

23. Fine S L, Martin D F, Kirkpatrick P. Pegaptanib sodium.*Nat Rev Drug Discov.* 2005;4:187–188. [PMID: 15770779 DOI: 10.1038/nrd1677]

24. Kang C. Avacincaptad Pegol: First Approval.*Drugs.* 2023;83:1447–1453. [PMID: 37814173 DOI: 10.1007/s40265-023-01948-8]
